# A Biochemiluminescent Sialidase Assay for Diagnosis of Bacterial Vaginosis

**DOI:** 10.1038/s41598-019-56371-5

**Published:** 2019-12-27

**Authors:** Shengjun Wu, Xuexiang Lin, Kwok Min Hui, Su Yang, Xuanlan Wu, Yichen Tan, Meimei Li, Ai-Qing Qin, Qingxi Wang, Qi Zhao, Pengfei Ding, Kaisheng Shi, X. James Li

**Affiliations:** 1 Sir Run Run Shaw Hospital, Zhejiang University School of Medicine, Hangzhou, 310016 China; 2 Cellex (Shenzhen), Incorporated, Shenzhen, 518081 China; 3 Cellex, Incorporated, Research Triangle Park, NC 27709 United States; 4 Jinan Central Hospital, Jinan, 250013 China; 5 Yuhuangding Hospital, Yantai, 264000 China; 6 College of Automation, Hangzhou Danzi University, Hangzhou, 310018 China; 7 Shenzhen College of Advanced Technology, University of Chinese Academy of Sciences, Shenzhen, 518055 China

**Keywords:** Urogenital reproductive disorders, Glycobiology

## Abstract

Bacterial vaginosis (BV) is a common condition among women of reproductive age. A sensitive, quantitative and rapid assay is needed for the diagnosis of and, particularly, therapy monitoring for BV. Bacterial sialidase appears to play an important role in bacterial biofilms on vaginal epithelium, a condition closely associated with BV. Here, we report a biochemiluminescent sialidase assay that uses a substrate derivatized with firefly luciferin. In the presence of sialidase in the reaction, the substrate is cleaved to release luciferin, which is subsequently oxidized by firefly luciferase to generate a light signal. Thus, the light signal intensity can be used to detect and measure the relative concentration of sialidase in a vaginal sample as a means of BV diagnosis. All reagents are present in a reagent bead and sample buffer, enabling essentially a one-step assay. The assay is highly sensitive and quantitative, with a sensitivity and specificity of 95.40% and 94.94%, respectively, compared to the Amsel method. Interestingly, only 27.6% of those with BV had high levels of sialidase activity with a signal to cutoff ratio of 10 or more. The assay may be used for diagnosis of BV, risk assessment of BV patients in terms of sialidase activity levels, and monitoring antibiotic therapy.

## Introduction

Bacterial vaginosis (BV) is a common condition^1–6^. Globally, 20–30% of women of reproductive age suffer from BV, a condition that is associated with an increased chance of preterm abortion, pelvic inflammatory disease (PID), sexually transmitted diseases including infection of human immunodeficiency virus (HIV), and even obesity^7–12^. Despite numerous efforts, the exact etiology agent or condition(s) for BV is still not well understood. It is commonly recognized that BV results from a change in vaginal conditions, which promote the growth of anaerobic bacterial species, thereby leading to a shift from aerobic bacterial flora to anaerobic bacterial flora in the vagina. The change in vaginal bacterial flora is often, but not always, associated with symptoms such as gray to white discharge, “fishy” smell upon addition of alkali solution, elevated pH (pH > 4.5), and epithelial cells heavily coated with bacteria, which are known as the Amsel criteria for BV^13^.

The healthy vaginal flora consists of predominantly (90–95%) *Lactobacillus spp*^5^. The most commonly cited microorganism in a BV flora is the anaerobic bacterium *Gardnerella vaginalis*, although other species are also found in BV flora^14^. *G. vaginalis* was found in culture samples from nearly all symptomatic women with BV but only in approximately half of healthy women. One proposed etiological role of *G. vaginalis* in BV is that this bacterial species first establishes a biofilm on vaginal epithelium^15,16^. Biofilm is critical for the survival of *G. vaginalis* in the vagina as it creates an anaerobic environment, which in turn invites other anaerobic bacterial species.

Sialidase appears to play an important role in biofilm formation by removing the polysaccharide substances on vaginal epithelium cells. There are several lines of evidence supporting this hypothesis. Much higher sialidase activity was detected in vaginal samples from women with BV^17,18^. Sialidase has been associated with biofilm formation in the vagina^19–22^. The two sialidase enzymes encoded by the genes NanH2 and NanH3 account for a wide spectrum of substrate preferences, including α2–3- and α2–6-linked *N*- and *O*-linked sialoglycans^23^. Third, a study using HeLa cervical epithelium cells showed that the sialidase inhibitor zanamivir, which inhibited 30% of the whole *G. vaginalis* cell sialidase activities, reduced epithelium cell association with and invasion by *G. vaginalis* by 50%, despite the fact that zanamivir was designed to inhibit influenza viral neuraminidase and not bacterial sialidase^24^. Finally, high levels of sialidase activity were associated with preterm birth risk, one of the risks associated with BV^18^.

Naturally, sialidase activity in vaginal samples has also been used as a diagnostic marker for BV^25^. A sialidase substrate, which can lead to color change upon cleavage by sialidase, was shown to have high predictive value for the diagnosis of BV^25^. Because of its simplicity, this method is widely used as a point of care BV test. However, the main drawbacks of this colorimetric assay are that it lacks sensitivity and is a qualitative assay, which limits its utility for certain applications that depend on a quantitative signal^26,27^. For example, a quantitative sialidase activity assay may be used to stratify BV patients according to the levels of sialidase activity, which may in turn be used to assess the other risks associated with BV. A more sensitive and quantitative assay may also be used for monitoring the efficacy of an antibiotic therapy.

Here, we report a luciferase-based biochemiluminescence assay, the qBV assay, for the detection of sialidase activity in vaginal samples and its use for the diagnosis of BV. This assay is a quantitative assay, with its signal proportional to the sialidase activity in a sample. Thus, it may potentially be used to diagnose BV and stratify patients with BV according to sialidase activity.

## Results

### Assay principle

**Figure 1:**
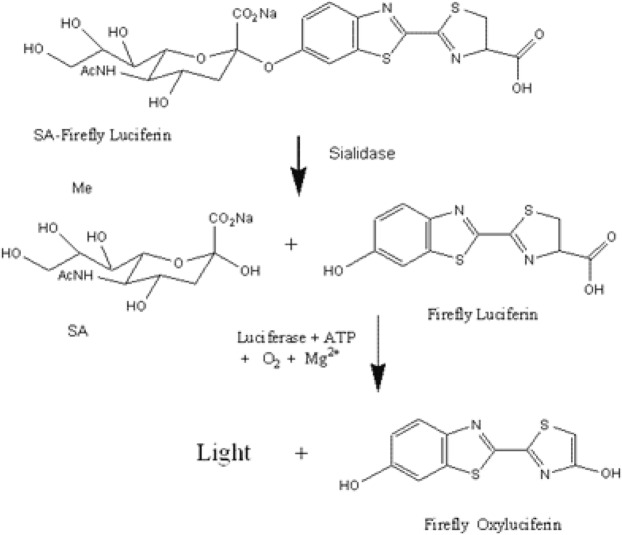
A diagram showing the reaction scheme of the qBV assay. Sialidase cleaves the substrate sialic acid *– O* –luciferin (SA-Firefly Luciferin) to release luciferin, which is subsequently oxidized by luciferase in the reaction to produce a detectable light signal. The light signal indicates the presence of sialidase in the sample.

The assay principle of the qBV assay is illustrated in Fig. 1. The qBV assay uses a sialidase substrate, sialic acid – *O* – firefly luciferin (SA-*O*-luciferin). In the presence of sialidase in a reaction, the substrate is cleaved to free luciferin, which becomes an active substrate of firefly luciferase. In the presence of firefly luciferase, the free luciferin is oxidized to oxyluciferin, resulting in a visible light signal that can be detected with a luminometer.

The assay involves two enzymatic reactions, the sialidase and luciferase reactions. To simplify the assay, a master mix was formulated such that the two reactions could occur in the same reaction mixture. With this single reaction mix format, the presence of sialidase in the reaction leads to the production of a continuous light signal, which is known as a real-time reaction. When the light signal intensity of the luciferase reaction is dependent on the product of the sialidase reaction, *i.e*., luciferin, the signal intensity is expected to be proportional to sialidase activity, thereby enabling quantitative detection of the sialidase activity in the sample.

### Substrate suitability

The substrate SA-*O*-luciferin was synthesized in-house. The synthetic substrate has two possible conformational isomers with regard to the *O*-glycosidic bond between sialic acid and luciferin, the α- and β- linkage. Since most forms of the *O*-glycosidic linkage between sialic acids and the cell membrane are α-linkage^28,29^, the sialidases from vaginal bacterial species are expected to prefer a substrate with an α-linkage. To determine how efficiently the substrate could be hydrolyzed by a sialidase that is known to favor a substrate with α-linkage, we used an influenza viral neuraminidase, which is also known as sialidase, to determine the appropriateness of the substrate^30^. Influenza viral neuraminidases are well characterized, and recombinant influenza viral neuraminidases are readily available. We used a recombinant influenza viral neuraminidase derived from an avian influenza virus (H6N2) for this experiment.

**Figure 2:**
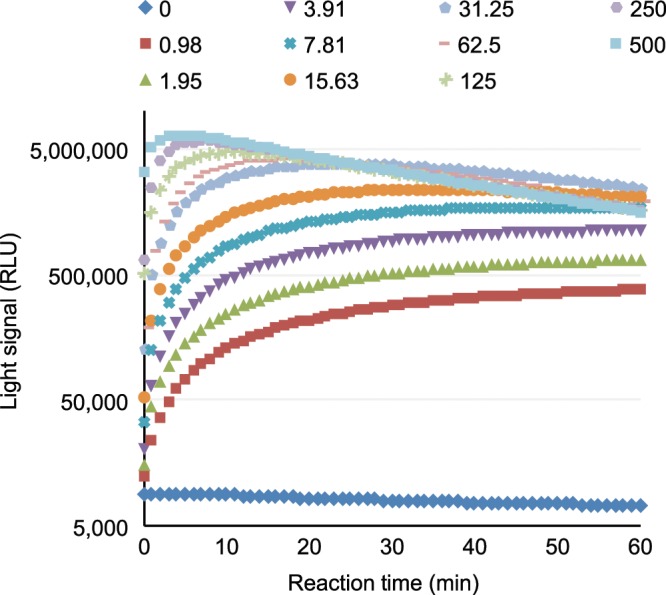
Reaction kinetics of recombinant influenza viral neuraminidase. Serially diluted recombinant influenza viral sialidase was mixed with the master mix and immediately placed in a luminometer. Light signals were collected over a period of 60 minutes. The numbers on top of the figure denote the amounts of input influenza neuraminidase (pg/0.25 mL reaction).

**Figure 3:**
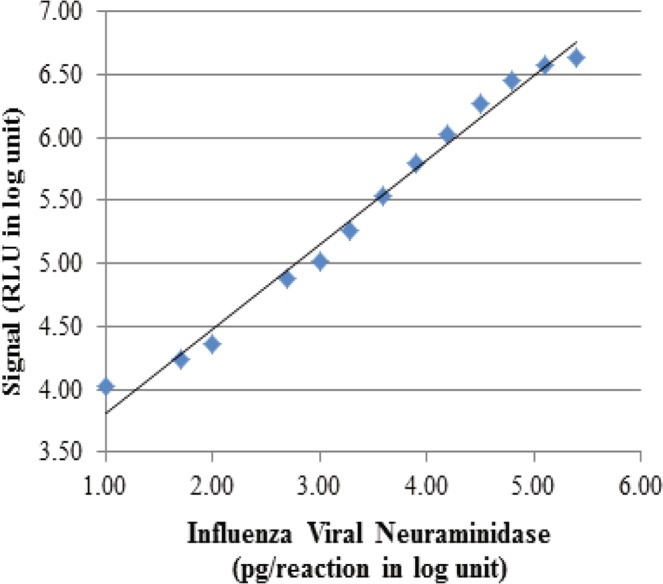
Linearity and linear range of influenza viral neuraminidase. Four replicates of serially diluted recombinant influenza viral neuraminidase were mixed with the master mix, incubated at room temperature for 5 minutes, and measured for light signal output. The mean signals from the 4 replicates were plotted against the input neuraminidase amounts. See Table 1 for raw data.

Various concentrations of the recombinant influenza viral neuraminidase were diluted into a master mix solution, which contained all components necessary to enable both neuraminidase/sialidase and luciferase reactions, and immediately placed in a luminometer to measure the light signal over a period of 60 minutes. The signal rapidly increased in the first 10 minutes of the reaction, followed by a plateau that lasted at least another 50 minutes (Fig. 2). At low to medium concentrations of the enzyme, the plateau could last more than 3 hours (data not shown). A slightly reduced signal was observed at the latter half of the plateau period when high concentrations of neuraminidase were present in the reaction (Fig. 3), likely due to substrate depletion. Thus, 5–20 minutes after reaction initiation appears to be the optimal time to collect a single measurement of the signal.

To estimate the detection limit and linear range of the assay, four replicates of each sample with varying influenza neuraminidase concentrations in the master mix were incubated for 5 minutes, and their light output was measured for 10 seconds. The assay exhibited a linear range between 10 and 250,000 pg/reaction influenza viral neuraminidase (R^2^ = 0.986; Fig. 3).

The mean, coefficient of variation and signal to noise ratio were calculated for different concentrations of neuraminidase and are presented in Table 1. The assay was highly sensitive, with 10 pg/reaction as the lower limit of detection (Table 1). These results showed that the substrate was suitable for use in the detection of sialidase that favors α-linkage of the *O*-glycosidic bond between sialic acid and luciferin.

**Table 1:** Results from four replicates of influenza viral neuraminidase at various concentrations using the master mix formulation.

Input enzyme (pg/reaction)	Mean RLU (n = 4)	% CV	S/N
250,000	4,314,512 ± 98,412	2.28	569.90
125,000	3,725,267 ± 227,959	6.12	492.07
62,500	2,860,471 ± 178,124	6.23	377.84
31,250	1,821,260 ± 38,316	2.10	240.57
15,625	1,068,100 ± 88,771	8.31	141.09
7,800	633,784 ± 53,695	8.47	83.72
3,900	346,125 ± 31,569	9.12	45.72
1,950	182,087 ± 17,462	9.59	24.05
1,000	104,170 ± 7,486	7.19	13.76
500	76,776 ± 2,373	3.09	10.14
100	23,088 ± 1,308	5.66	3.05
50	17,051 ± 1,093	6.41	2.25
10	10,499 ± 1,108	10.56	1.39
0	7,571 ± 183	2.42	1.00

The signal to noise (S/N) ratio was calculated by dividing the signal from reactions containing the input enzyme with the background signal of the reaction without input enzyme. RLU: relative light units. CV: coefficient of variation.

### The qBV assay

The assay formulation for the influenza neuraminidase detection described above was too sensitive for detection of sialidase in vaginal samples as vaginal samples from even healthy women are expected to have some level of sialidase. Thus, a less sensitive version of the assay was formulated and lyophilized as reagent beads, which are referred to as the qBV assay. For the prospective clinical study, one reagent bead was used for one sample testing. When a reagent bead is suspended in 0.25 mL of sample buffer, the solution has all the components necessary for both the sialidase and luciferase reactions, albeit with less sensitivity.

**Figure 4:**
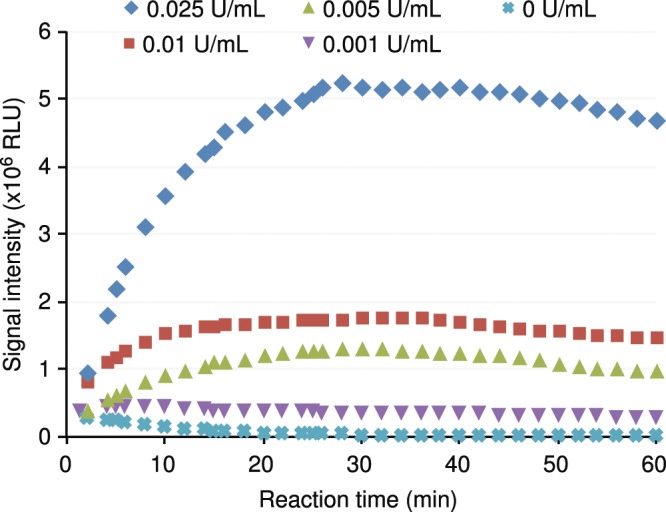
Reaction kinetics of a bacterial sialidase. Samples containing diluted bacterial sialidase were added to a qBV reagent bead and immediately placed in a luminometer. Light signals were collected over a period of 60 minutes.

To evaluate the reaction kinetics of this formulation, diluted samples of the BV Blue Control were used for this experiment. Since the certificate of analysis listed the concentration as >10 U/40 µL (or “>250 U/mL”), the BV Control was diluted by 250,000, 50,000, 25,000, or 10,000 times, corresponding to sialidase concentrations of >0.001, >0.005, >0.01, or >0.025 U/mL. Two hundred fifty (250) microliters of these samples, including a negative sample, was added to a reagent bead, followed by signal collection for up to 60 minutes. For lower concentrations of sialidase, the signal reached a plateau at approximately 10 minutes, whereas the signal did not reach a plateau until approximately 25 minutes for a sample with a high sialidase concentration (Fig. 4). Information on other performance characteristics of the qBV assay is provided in the Supplemental Information.

**Figure 5:**
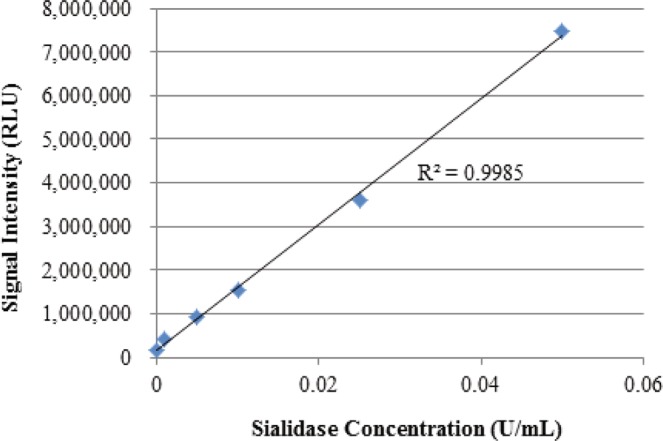
Linearity and linear range of the qBV assay. Four replicates of serially diluted bacterial sialidase were mixed with a qBV reagent bead, incubated at room temperature for 5 minutes, and measured for light output. The mean signals from the 4 replicates were plotted against the input sialidase concentrations. See Table 2 for raw data.

A rapid bacterial vaginosis test is preferred for diagnostic use. Thus, signal collection at 10 minutes was used in a diagnostic assay. To evaluate this method, four replicates of samples containing sialidase concentrations ranging from 0 to >0.05 U/mL were tested. The signal was collected at 10 minutes. As shown in Table 2, the coefficient of variation (% CV) ranged from 4.13% to 6.72%. The mean test results were plotted against the sialidase concentrations. There was a linear relationship between the signal intensity and sialidase concentrations up to 0.05 U/mL (Fig. 5).

**Table 2:** Test results of serially diluted bacterial sialidase.

Bacterial Sialidase (U/mL)	Mean RLU (n = 4)	% CV	S/CO
0.1	9,823,395 ± 548030	5.58	24.56
0.05	7,475,201 ± 502629	6.72	18.69
0.025	3,591,607 ± 202505	5.64	8.98
0.01	1,551,421 ± 93932	6.05	3.88
0.005	920,349 ± 48170	5.23	2.30
0.001	426,777 ± 28021	6.57	1.07
0	157,204 ± 6495	4.13	0.39

Four replicates of each dilution, including a negative sample without sialidase, were tested using the qBV reagent. RLU: relative light units. CV: coefficient of variation. S/CO: signal to cutoff ratio.

### Clinical evaluation

To evaluate the performance of the qBV assay in clinical settings, a prospective clinical study was conducted. A total of 423 participants in three clinical sites were enrolled in the study. A vaginal swab sample was first collected and stored at 2–8 °C from an enrollee for the qBV assay. The enrollee was then subjected to BV diagnosis by clinicians using the Amsel’s method. According to the Amsel’s criteria, BV was diagnosed when three of the four diagnostic criteria were present: (1) a vaginal fluid pH > 4.5; (2) >20% of epithelial cells are “clue” cells; (3) milky homogenous, adherent vaginal discharge; and (4) a positive “whiff” test, which is an amine or “fishy” odor noted after the addition of 10% alkaline solution. The clinical diagnosis with the Amsel’s method was used as the gold standard for comparison.

The vaginal swab was eluted in 1.0 mL of sample buffer for the qBV assay. A total of 250 microliters of the resulting sample was added to a qBV reagent bead. The signal intensity was measured with a luminometer after incubation at room temperature for 10 minutes. The qBV test result interpretation was based on a cutoff value of 400,000 relative light units (RLU) when a Helios 2000 luminometer was used, which was previously determined.

The results are presented in Table 3. Of the 423 participants, eighty-seven (87) were diagnosed with BV by the Amsel method. Of these 87 participants who had BV by clinical methods, eight-three (83) were diagnosed with BV with the qBV assay. The BV rate in this population was 20.57% by the Amsel method and 19.62% by the qBV assay.

**Table 3:** Test results from the prospective clinical study.

		BV Confirmed by Amsel		
		Positive	Negative	Total
BV Confirmed by qBV Assay	Positive	83 (TP)	17 (FP)	100
	Negative	4 (FN)	319	323
	Total	87	336 (TN)	**423**

The gold standard method used in the study was the Amsel method. The sensitivity and specificity were estimated by comparing the test results of the qBV assay with those of the gold standard method (the Amsel method). TP: true positive; FP: false positive; TN: true negative; FN: false negative.

Overall, the qBV assay had a sensitivity and specificity of 95.40% (95% confidence interval: 90.79–100%) and 94.94% (95% confidence interval: 92.47–97.41%), respectively. The positive predictive value (PPV) and negative predictive value (NPV) were 83% and 98.76%, respectively. The prevalence of BV in Asian women is often cited as 20–30%. Based on an assumed BV prevalence of 25% in the study populations and using the likelihood ratio method, the positive posttest probability is estimated to be 86.27% when the test result is positive.

**Figure 6:**
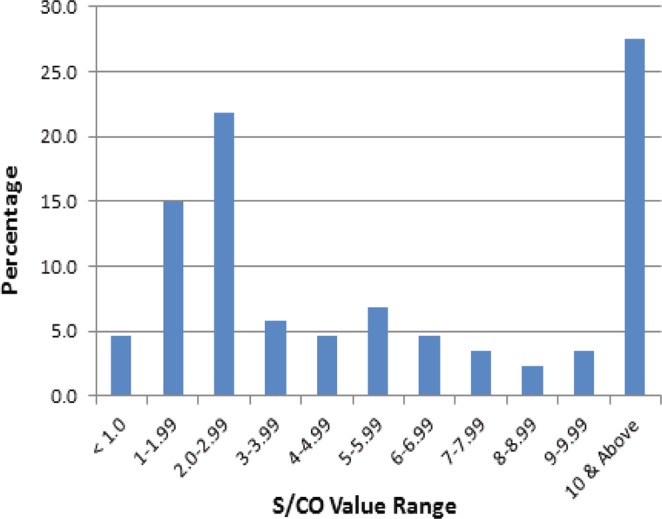
Histogram of the sialidase activity in terms of signal to cutoff values (S/CO) among participants diagnosed with BV.

The S/CO values are indicative of the levels of sialidase activity in the samples. The 87 BV-positive samples by the Amsel method are grouped according to their S/CO values by the qBV assay. Of these 87 BV-positive samples, 4.6% had a qBV signal to cutoff value (S/CO) below 1.0, which was considered negative by the qBV assay, 47.1% had a low S/CO (1.0 to 4.99), 20.7% had a medium S/CO (5.0–9.99), and 27.6% had high S/CO values (equal to or greater than 10.0) (Fig. 6). These data showed that there were diverse levels of sialidase activity in the patients who were diagnosed with BV, and only 27.6% had high levels of sialidase activity.

## Discussion

BV is commonly encountered among women of reproductive ages. It is associated with many adverse health problems. It is well recognized that BV is a recurrent and difficult-to-treat condition. Improvement in the treatment outcome of BV requires a better understanding of the etiology of this condition, which should in turn lead to better diagnostic and treatment monitoring methods. Many studies have shown that the predominance of *G*. *vaginalis* species in the vagina is associated with BV. Evidence suggests that bacterial biofilm formation on the vaginal epithelium is associated with BV. Like many features of BV, it is not clear whether it is a BV condition that leads to bacterial biofilm formation, or a bacterial biofilm that leads to BV conditions. However, the difficult-to-treat feature of BV may be explained by bacterial biofilm formation, which is known to be less responsive to antibiotic therapies^31^.

Sialidase secreted from bacterial species appears to play an important role in biofilm formation in BV^17–22^. A more recent study showed that high concentrations of the sialidase gene containing *G*. *vaginalis* species in vaginal samples were associated with biofilm formation^22^. The qBV test is a rapid BV test that can also determine the levels of sialidase activities in the sample. Given the association of sialidase activity with biofilm formation, BV conditions and certain BV-associated risks, such as preterm birth, this assay may be a useful tool for use in BV diagnosis.

The qBV assay uses a firefly luciferin derivatized with a sialic acid moiety for detection of sialidase activity. In the presence of sialidase in the reaction, the substrate is cleaved to release luciferin, which becomes the substrate of luciferase. The qBV assay is a real-time assay in which cleaved luciferin is immediately consumed by luciferase to produce a light signal as luciferin is freed from the substrate through cleavage by sialidase. A real-time assay is known to be more quantitative. Indeed, the qBV assay is highly linear between S/CO values of less than 1.0 (none BV) and 18.75 (Fig. 5).

The test results of the qBV assay correlated well with those of the Amsel method. When compared to the Amsel method, the qBV assay had 95.40% and 94.94% sensitivity and specificity, respectively (Table 3). However, the qBV assay uses a simple luminometer and takes only 10 minutes, which makes BV diagnosis less cumbersome and more rapid. The quantitative nature of the assay may be used to stratify BV patients in terms of the sialidase activity levels. In the prospective study, approximately half (47.1%) of the BV-positive patients by the qBV assay had low levels of sialidase activity in vaginal samples with signal to cutoff values of less than 5.0, whereas 27.6% had high sialidase activity with signal to cutoff values of 10 or more.

Because of its quantitative nature, the assay may be used to stratify BV patients in certain populations. For example, it has been previously shown that the level of sialidase activity correlated well with the risk of preterm birth among pregnant women^18^. Thus, the qBV assay may be used as a prognostic tool for assessing the risk of preterm birth and monitoring therapy. A large clinical study showed that only high-risk pregnancies benefited from antibiotic treatment^32^. The identification of high-risk pregnancies among women with BV requires an assay that is easy to use, inexpensive, rapid and suitable for risk assessment.

## Methods

### Materials

Recombinant influenza viral neuraminidase (sialidase) was obtained from the Biodefense and Emerging Infections Research Resources Repository (www.beiresources.org). It was a full-length clone of an N2 gene from an avian influenza virus, A/Shorebird/Delaware/127/97 (H6N2), expressed in the Sf9 insect cells using a baculovirus vector. Molar concentrations of recombinant influenza viral neuraminidase were calculated using a tetrameric molecular weight of 240 kDa. The bacterial sialidase was from *Clostridium perfringens*. BSA, DDT coenzyme A, and sodium ATP were purchased from Sigma or Fisher Scientific. The BV Blue control was purchased from Gryphus Diagnostics, LLC (Knoxville, TN).

### Synthesis of the substrate

Methyl (5-acetamido-4,7,8,9-tetra-*O-*acetyl-3,5-dideoxy-D-glycero-b-D-alactononulopyranosyl chloride) Onate was prepared in two steps from a commercially available sialidic acid as described elsewhere (18). The crude chloride was purified by a silica gel plug and eluted with 200 mL of 80–90% EtOAc in hexane. After concentrating the filtrate, the chloride was obtained as a white powder and immediately used for the next coupling reaction.

Luciferin (0.83 g) and the phase transfer catalyst tetrabutylammonium hydrogen sulfate (2.43 mmoles) were placed in a 100-mL round-bottomed flask and treated with 12.5 mL of CH_2_Cl_2_ and 17.5 mL of 0.5 N NaOH at room temperature forming a two-phase mixture. A total of 1.24 grams of the chloride prepared as described above was dissolved in 5 mL of CH_2_Cl_2_ and added to the mixture. After an hour of vigorous stirring, the reaction mixture was diluted with CH_2_Cl_2_ and loaded into a separatory funnel that contained saturated sodium bicarbonate solution. After the organic layer was separated, the aqueous layer was further extracted twice with CH_2_Cl_2_. The combined organic layers were washed with H_2_O and dried over anhydrous Na_2_SO_4_. The organic solution was treated with 10 drops of Et_3_N and concentrated. The crude product was purified by silica gel chromatography.

The resulting pyranoside was deprotected at 0 °C for 5 minutes in a mixture of 6.5 mL of THF, 6.5 mL of MeOH and 12 mL of 1 N NaOH. The mixture was further stirred at room temperature for an hour, followed by the addition of 1.05 g of solid sodium bicarbonate to lower the pH. The solution was filtered using a Buchner funnel, rinsed with a small volume of water, and purified by reverse-phase preparative HPLC. The fractions containing the final product were pooled and lyophilized. Based on our preliminary studies, two rounds of HPLC-based purification are necessary to completely remove both contaminating components and free luciferin.

### qBV assay

The qBV assay refers to the one-step assay in which both sialidase and luciferase reactions occur in a single reaction. The qBV assay was prepared in two forms: 2x master mix solution format and lyophilized reagent format. The 1x master mix contained the following: 50 mM HEPES (pH 7.0–7.2), 0.5% BSA, 4 mM ATP, 10 mM DTT, 3.3 mM coenzyme A, 15 mM magnesium sulfate, 4 mM calcium chloride, 1 mM substrate and 100 mg/L luciferase. A 2x concentrated master mix solution was prepared for the studies using the influenza viral neuraminidase.

The lyophilized form of the qBV reagent was used in the prospective clinical study and was prepared as follows: thirty (30) microliters of a concentrated master mix solution containing 1% BSA, 3 mM ATP, 10 mM DTT, 1 mM coenzyme A, 1 mM substrate and 4 micrograms/mL luciferase was lyophilized as a reagent bead. In addition, a sample buffer containing 50 mM HEPES (pH 7.0–7.2), 15 mM magnesium sulfate, 4 mM calcium chloride, 0.5% BSA, 0.5% Triton X-100, and 0.1% Proclin 950 was prepared. One reagent bead was used for one sample assay. To perform a qBV assay for a clinical sample, a vaginal swab was suspended in 1 mL of sample buffer, and two hundred fifty (250) microliters of the resulting sample was added to a reagent bead. After incubation at room temperature for 10 minutes, the test tube was placed in a Helios 2000 luminometer to measure the light signal. BV is diagnosed when the signal is at or greater than the cutoff value of 400,000 relative light units (RLU).

### Collection of clinical specimens

From April 2017 to May 2019, 423 participants from three sites were enrolled in the study. The study was approved by the medical ethics committee at all three clinical sites (Shandong University Jinan Central Hospital Medical Ethics Committee, the National Clinical Trial Network Medical Ethics Committee at Yantai Yuhuangding Hospital, and the Medical Ethics Committee of Zhejiang University Sir Run Run Shaw Hospital). Informed consent was obtained from each participant in two of the three sites (Jinan Central Hospital and Yantai Yuhuangding Hospital), which together collected 221 samples. A vaginal swab sample was collected from each participant and stored at 2–8 °C for qBV testing. The participant was subject to BV diagnosis with the Amsel method. The study at two of the three sites (Shandong University Jinan Central Hospital and Zhejiang Sir Run Run Shaw Hospital) was designed and conducted according to the Declaration of Helsinki and other requirements of the China Food and Drug Administration. Two hundred and two (202) leftover samples were used in the third site (Zhejiang University Sir Run Run Shaw Hospital), whose medical ethics committee approved the study without informed consent requirement. The stored samples for qBV testing were normally tested in batches at the end of each day. All methods were carried out according to relevant guidelines and regulations.

## Supplementary information

Supplementary information 
