# Supplementary material for: A Biochemiluminescent Sialidase Assay for Diagnosis of Bacterial Vaginosis
Source: Sci Rep. 2019 Dec 27;9:20024. doi: 10.1038/s41598-019-56371-5 (PMC6934538; doi:10.1038/s41598-019-56371-5)
Supplement: Supplementary file 1 — Supplementary information [file 41598_2019_56371_MOESM1_ESM.docx]

**Supplementary Information**

**A Biochemiluminescent Sialidase Assay for Diagnosis of Bacterial Vaginosis**

Shengjun Wu^1^, Xuexiang Lin^2,7^, Kwok Min Hui^2,3^, Su Yang^1^, Xuanlan Wu^1^, Yichen Tan^2^, Meimei Li^1^, Ai-Qing Qing^2^, Qingxi Wang^4^, Qi Zhao^5^, Pengfei Ding^6^, Kaisheng Shi^2^, X. James Li^2,3^

^1^Sir Run Run Shaw Hospital, Zhejiang University School of Medicine, Hangzhou, 310016, China

^2^Cellex (Shenzhen), Incorporated, Shenzhen, 518081, China

^3^Cellex, Incorporated, Research Triangle Park, NC 27709, USA

^4^Jinan Central Hospital, Jinan, 250013, China

^5^Yuhuangding Hospital, Yantai, 264000, China

^6^College of Automation, Hangzhou Danzi University, Hangzhou, 310018, China

^7^Shenzhen College of Advanced Technology, University of Chinese Academy of Sciences, Shenzhen, 518055, China

**Reproducibility**

To evaluate the within lot reproducibility of the qBV assay, ten (10) replicates of a sample spiked with a positive control containing a bacterial sialidase were tested with each of three lots of reagents. The results are presented in **Table S1**. The coefficients of variation ranged from 2.13 to 8.96%.

**qBV Reagent Stability**

**Refrigeration storage condition (2-8^o^C)**. Three reagent lots were stored at refrigerated conditions over a period of 12 months. A reagent bead was placed in a glass test tube. Multiple test tubes containing reagent beads were then packaged in moisture proof pouches. For this stability study, opened or unopened pouches were stored in a refrigerator. The reagent tubes were removed from the pouches and tested at time 0, 1 month, 6 months or 12 months. No deterioration was observed over this storage period (**Table S2**). Thus, the reagent is stable at 2-8^o^C for at least 12 months.

**Ambient storage condition (20-30^o^C).** Three reagent lots were stored at ambient conditions in sealed pouches. The reagent tubes were removed and tested at day 0, 7, 14 and 30. After each use, the pouches were resealed. No significant deterioration was observed up to 14 days under this storage condition. However, storage period longer than 14 days resulted in significant reduction in signal to noise ratio (**Table S3**). The reduction in signal to noise ratio was due to the increase in background signal, indicating that the substrate might under autolysis at elevated storage temperature. Thus, the reagent is stable at ambient condition for up to 14 days.

**Elevated temperature storage condition (37^o^C).** Three reagent lots were stored at 37^o^C oven in sealed pouches. The reagent tubes were removed and tested at day 0, 3, 7, 10, 14, 17, 21, 24 and 30. After each use, the pouches were resealed. No significant deterioration was observed up to 3 days under this storage condition, after which significant reduction in signal to noise ratio was observed (**Table S4**). The reduction in signal to noise ratio was due to the increase in background signal. Thus, the reagent is stable at 37^o^C for up to 3 days.

**Potential Interference Factors**

When tested at a concentration of up to 2×10^6^ CFU/mL, the following bacterial species exhibited no interference on the assay: *Enterobacter cloacae*, *Enterococcus sp*., *Escherichia coli*, *Klebsiella pneumoniae*, *Pseudomonas aeruginosa*, *Salmonella*, *Serratia marcescens*, *Staphylococcus aureus*, *Streptococcus agalactiae*, *Streptococcus pyogenes*, and *Candida albicans*.

The following chemicals exhibited no interference on the assay when tested at the indicated concentration: EDTA (0.25%), mucin (0.25%), phenylephrine (0.1%), oxymetazoline (0.005%), dexamethasone (0.5 mg/mL), Flunisolide (0.5mg/mL), beclomethasone (0.5mg/mL), triamcinolone (0.5mg/mL), menthol (0.5mg/mL), tobramycin (0.5mg/mL) , and benzocaine (0.05mg / mL).

Presence of fluticasone (0.5mg/mL) or sodium chloride (>10%) in the sample reduced the signal and therefore may result in false negative test result.

**Tables for Supplementary Information**

| Replicate | Signal (RLU) | | |
| --- | --- | --- | --- |
|  | Lot 1 | Lot 2 | Lot 3 |
| 1 | 6704681 | 7024619 | 10587252 |
| 2 | 6902158 | 7142838 | 9765788 |
| 3 | 7003541 | 7265463 | 11235679 |
| 4 | 6782354 | 6847665 | 9976547 |
| 5 | 6496738 | 6796700 | 12800137 |
| 6 | 6706542 | 7108110 | 10437898 |
| 7 | 6825001 | 6796560 | 9796488 |
| 8 | 6894073 | 6908450 | 11224063 |
| 9 | 6656041 | 7065431 | 11124654 |
| 10 | 6738269 | 6877479 | 9806110 |
| Average | 6770940 | 6983331 | 10675461 |
| SD | 144348 | 161240 | 956811 |
| %CV | 2.13 | 2.31 | 8.96 |

**Table S1**: Reproducibility Test Results. Ten (10) replicates were tested with each of three reagent lot using a positive control. Average, standard deviations (SD) and coefficients of variation (CV) were calculated from the 10 replicate test results.

| Month | Sealed Pouch  (S/N) | | | | Opened Pouch  (S/N) | | | |
| --- | --- | --- | --- | --- | --- | --- | --- | --- |
|  | Lot 1 | Lot 2 | Lot 3 | Average  (n=3) | Lot 1 | Lot 2 | Lot 3 | Average  (n=3) |
| 0 | 386 | 478 | 206 | 357 ± 138 | 365 | 141 | 1,000 | 502 ± 445 |
| 1 | 348 | 256 | 305 | 303 ± 46 | 350 | 186 | 143 | 226 ± 109 |
| 6 | 444 | 421 | 459 | 442 ± 19 | 471 | 421 | 485 | 459 ± 33 |
| 12 | 500 | 485 | 471 | 485 ± 15 | 486 | 516 | 370 | 457 ± 77 |

**Table S2**: Reagent Stability When Stored at Refrigerated Conditions. Reagent beads were placed in test tube vials, stored in opened or sealed pouches and tested at month 0, 1, 6 and 12. No deterioration of signal to noise ratio was observed in either sealed or opened pouches and up to 12 months. S/N: signal to noise ratio.

| Day | Signal to Noise Ratio | | | |
| --- | --- | --- | --- | --- |
|  | Lot 1 | Lot 2 | Lot 3 | Average  (n=3) |
| 0 | 291 | 354 | 330 | 325 ± 31 |
| 7 | 341 | 363 | 361 | 355 ± 12 |
| 14 | 267 | 256 | 304 | 276 ± 23 |
| 30 | 132 | 128 | 87 | 115 ± 25 |

**Table S3**: Reagent Stability When Stored at Ambient Conditions. Reagent beads were placed in test tube vials, packaged in sealed pouches, stored at ambient conditions and tested at day 0, 7, 14 and 30. No significant deterioration of signal to noise ratio was observed up to 14 days.

| Day | Signal to Noise Ratio | | | |
| --- | --- | --- | --- | --- |
|  | Lot 1 | Lot 2 | Lot 3 | Average  (n=3) |
| 0 | 154 | 229 | 276 | 220 ± 62 |
| 3 | 141 | 181 | 224 | 182 ± 42 |
| 7 | 93 | 121 | 137 | 117 ± 22 |
| 10 | 81 | 84 | 55 | 73 ± 16 |
| 14 | 61 | 100 | 78 | 80 ± 20 |
| 17 | 67 | 65 | 61 | 64 ± 3 |
| 21 | 55 | 67 | 78 | 66 ± 11 |
| 24 | 64 | 50 | 65 | 60 ± 9 |
| 30 | 75 | 54 | 49 | 59 ± 13 |

**Table S4**: Reagent Stability When Stored at Elevated Temperature (37^o^C). Reagent beads were placed in test tube vials, stored in sealed pouches at ambient conditions and tested at days 0, 3, 7, 10, 14, 17, 21, 24 and 30. No significant deterioration of signal to noise ratio was observed up to 3 days.
